# The epidemiology ontology: an ontology for the semantic annotation of epidemiological resources

**DOI:** 10.1186/2041-1480-5-4

**Published:** 2014-01-17

**Authors:** Catia Pesquita, João D Ferreira, Francisco M Couto, Mário J Silva

**Affiliations:** 1 LASIGE, Campo Grande, Lisboa, Portugal; 2 INESC-ID, Av. Alves Redol, Lisboa, Portugal; 3 Universidade de Lisboa, Lisboa, Portugal

## Abstract

**Background:**

Epidemiology is a data-intensive and multi-disciplinary subject, where data integration, curation and sharing are becoming increasingly relevant, given its global context and time constraints. The semantic annotation of epidemiology resources is a cornerstone to effectively support such activities. Although several ontologies cover some of the subdomains of epidemiology, we identified a lack of semantic resources for epidemiology-specific terms. This paper addresses this need by proposing the Epidemiology Ontology (EPO) and by describing its integration with other related ontologies into a semantic enabled platform for sharing epidemiology resources.

**Results:**

The EPO follows the OBO Foundry guidelines and uses the Basic Formal Ontology (BFO) as an upper ontology. The first version of EPO models several epidemiology and demography parameters as well as transmission of infection processes, participants and related procedures. It currently has nearly 200 classes and is designed to support the semantic annotation of epidemiology resources and data integration, as well as information retrieval and knowledge discovery activities.

**Conclusions:**

EPO is under active development and is freely available at https://code.google.com/p/epidemiology-ontology/. We believe that the annotation of epidemiology resources with EPO will help researchers to gain a better understanding of global epidemiological events by enhancing data integration and sharing.

## Background

Epidemiology is the study of the factors influencing the occurrence and distribution of health-related states or events in specified populations, and the application of this knowledge to control health problems [1]. It is a multi-disciplinary subject that integrates diverse areas of knowledge, such as medicine, biology, statistics, social sciences and geography.

Epidemiology is becoming increasingly data-intensive, considering the large volumes of data generated by biomedical research and by the recent explosion of mobile phone and Internet usage - which contains epidemiologically relevant behaviors, such as disease symptoms reports [2], and also the data created by large-scale computational simulations and models of disease transmission and spread [3,4]. To handle these challenges, epidemiology needs to embrace the new scientific methodology designated as the fourth paradigm, whereby vast troves of data are collected, analyzed, validated and visualized [5]. Ontologies are crucial to support this new paradigm, since they provide the means to semantically describe epidemiological resources, supporting their categorization and sharing.

Consider the following example: a research team is building a model for herd immunity in populations where a measles vaccine can be administered. To achieve this, they need data on measles incidence rates and vaccination rates in different populations/locations over time, as well as other parameters, such as birth rate, factors influencing vaccination (e.g. legal frame, income and education level of parents), transmission mode and secondary attack rate (i.e. the number of cases of an infection that occur among contacts within the incubation period following exposure to a primary case in relation to the total number of exposed contacts). These data can then be used to fit the parameters of their model. Traditionally, to collect the data, researchers would conduct extensive literature searches to find a set of relevant scientific articles, read them to extract the relevant information and/or contact the authors to request access to the datasets directly. The epidemiology community has not yet adopted the practice of publicly sharing datasets in open databases [6], which further hinders the collection of pertinent data. However, epidemiology is a domain where timeliness is crucial. For instance, when facing a new pandemic, laboratories need to be able to produce new vaccines very quickly, and public health officials need to understand the disease and its spread so they can issue recommendations to the population to effectively contain the pandemic and diminish its impact. To make data collection more efficient and effective, epidemiological resources need to be easily searchable and retrievable, which can be achieved by semantic-enabled platforms for sharing epidemiological resources. An approach is supporting the annotation of datasets with ontological concepts, so that the semantics encoded in ontologies can be used to find relevant resources. For instance, resources that do not refer to measles, but to other typical childhood diseases with the same transmission mode can very well be of interest to extract parameters for the measles herd immunity model.

The only currently available ontology specifically intended for epidemiology is integrated into the BioCaster Global Health Monitor [7], a news filter created with the aim of providing “an early warning monitoring station for epidemic and environmental diseases”. However, the 2,000 classes of the BioCaster ontology are insufficient to provide enough coverage and granularity for a full semantic annotation of epidemiological resources. For instance, there is no class for vaccine, and diseases are direct instances of Human Disease or Avian Disease, which are direct subclasses of Disease, highlighting the complexity of modeling these domains [8]. However, in such a multidisciplinary domain as epidemiology, several key areas have already been described in existing ontologies, including, among others, the Disease Ontology [9], Infectious Disease Ontology (IDO) [10], Symptom Ontology [11], Vaccine Ontology [12] and the Pathogen Transmission Ontology (TRANS) [11]. In previous work, we have outlined a Network of Relevant Ontologies for Epidemiology (NERO) [13]. We found that while some concepts are fully covered by these ontologies, others are not, in particular the specific epidemiological concepts that are seldom used outside this domain, such as, for instance, parameters like ‘exposure ratio’ or ‘attack rate’. Consequently, a new ontology that covers these specific epidemiology concepts, while reusing and complementing relevant existing ontologies in related domains is needed. Bearing this in mind, we have created the Epidemiology Ontology (EPO), which aims at covering the areas of epidemiology not well described by other quality ontologies, particularly those related with metrics, parameters and models. EPO currently covers epidemiological and demographical parameters, for which there was very little coverage in surveyed ontologies, as well as transmission of infection, complementing classes from the TRANS ontology. In future versions, the scope of EPO will be expanded to include all parameters that influence epidemic processes, in articulation with existing and in development ontologies for public health and medical surveillance.

In this paper, we describe the current state of EPO and how it is related to other ontologies relevant for the epidemiological domain. We also explain how EPO is being used to annotate epidemiological resources in a platform for epidemiological resource sharing, where it supports data querying and integration, and provide examples of how it could also be used for annotation of other databases and literature. The current version of EPO has 190 classes, of which 118 are newly created and 33 are imported from two relevant OBO foundry candidate ontologies, IDO and TRANS. EPO uses the Basic Formal Ontology (BFO) [14] as an upper ontology, and IAO [15] as a source of annotation properties, further supporting its interoperability with other OBO foundry ontologies and candidate ontologies. We have submitted EPO to the OBO Foundry [16], as well as to the BioPortal site of the National Center for Biomedical Ontologies (NCBO) [17]. EPO is freely available at https://code.google.com/p/epidemiology-ontology/.

## Results

### Modelling

We used the Dictionary of Epidemiology (DoE) [1] in the creation the EPO. The Dictionary of Epidemiology is a well-established reference that captures the nomenclature commonly used in epidemiology. Most class labels, synonyms and definitions in EPO correspond to dictionary entries or sub-entries.

In the current version of EPO, we have focused our modeling activity in three major areas: demographic parameters, epidemiological parameters and transmission of infection.

**Figure 1:**
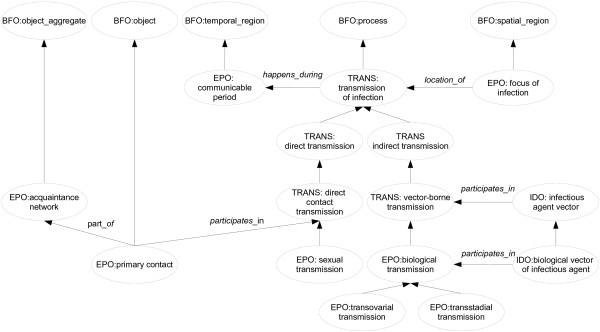
**A representative portion of EPO.** This diagram represents a portion of EPO and how EPO classes are related to each other and to other ontologies classes. Unlabeled arrows represent subclass relationships, and labeled arrows represent relations imported from RO. The ontology for each class is identified by its prefix.

Although some resources contain a few demographic parameters, such as MeSH [18] and NCI Thesaurus [19], we have found that the majority of such parameters are not represented in hierarchical vocabularies or ontologies. Likewise, the coverage for epidemiological parameters was also quite sparse. However, there are several resources that model transmission of infection, including the Pathogen Transmission Ontology (TRANS) with 25 classes fully dedicated to transmission of infection, the Host Pathogen Interaction Ontology [20], Influenza Ontology [21] and NCI Thesaurus. Nevertheless, TRANS models transmission of infection types only, and it does so in a different fashion from the DoE, with a different hierarchical organization and definitions. Consequently, we chose to include classes for transmission of infection in EPO in accordance with the entries in the DoE. Whenever an equivalent class was present in TRANS we imported it, but used the label and definition from the DoE as editor preferred label and definition, which resulted in reusing 14 TRANS classes, for a total of 21 transmission of infection types modeled in EPO. These classes are organized in single inheritance, in up to five levels, increasing the granularity level given by TRANS by two levels, but also widening its scope by including classes for the participants in the transmission of infection process. These include classes imported from IDO as well as EPO-specific classes, which are linked to their respective transmission type via *participates_in* relations (see Figure 1. for a relevant portion of EPO).

Furthermore, EPO also contains 17 classes dedicated to transmission of infection-related processes, such as isolation, containment and eradication, to name a few. These classes are particularly relevant for the description of public health procedures and their impact on epidemic events. Their articulation with transmission of infection types in describing epidemiological resources will allow the elucidation of the relations between these procedures and the mode of transmission.

In the demographic and epidemiological parameters branches we currently have 36 and 21 classes, respectively. These are organized in a multiple inheritance structure, with classes being both subclasses of either ‘demography parameter’ or ‘epidemiology parameter’ , as well as of their specific parameter type, like ‘rate’. To the best of our knowledge, there were no suitable ontologies from which to import classes in these areas, since the very few terms that exist are poorly defined and structured. However, we have included cross-references to relevant external resources, including the NCI Thesaurus, MeSH and SNOMED-CT [22]. One relevant aspect of these classes is that they allow the description of simulation experiments and models, which are increasingly being used by the epidemiology community, even during outbreaks and epidemics, to help understand the events and design response strategies. Annotations with EPO-defined parameters can directly support the reuse and meta-analysis of simulation results and models.

Tables 1 and 2 summarize the statistics on specific, imported and cross-referenced classes and properties.

**Table 1:** Statistics of EPO specific and imported classes and properties

**Ontology**	**Number of classes or properties**
Epidemiology Ontology (EPO)	118
Infectious Disease Ontology (IDO)	19
Pathogen Transmission Ontology (TRANS)	14
Basic Formal Ontology (BFO)	38
Relation Ontology (RO)	4
Information Artifact Ontology (IAO)	7
OBOInOWL	1
Phenotypic Quality Ontology (PATO)	1
Total	202

**Table 2:** Statistics on EPO cross-references

**Resource**	**Number of cross-references**
MSH	4
UMLS	1
NCI Thesaurus	9
SNOMED-CT	2
HPI	10
MDR	7
PATO	1
Total	34

### Examples

EPO currently covers three main branches: transmission mode, epidemiological parameters and demographic parameters. The transmission mode branch is highly interconnected with other ontologies, reusing many classes from IDO and TRANS. A snippet of this branch is depicted in Figure 1.

**Figure 2:**
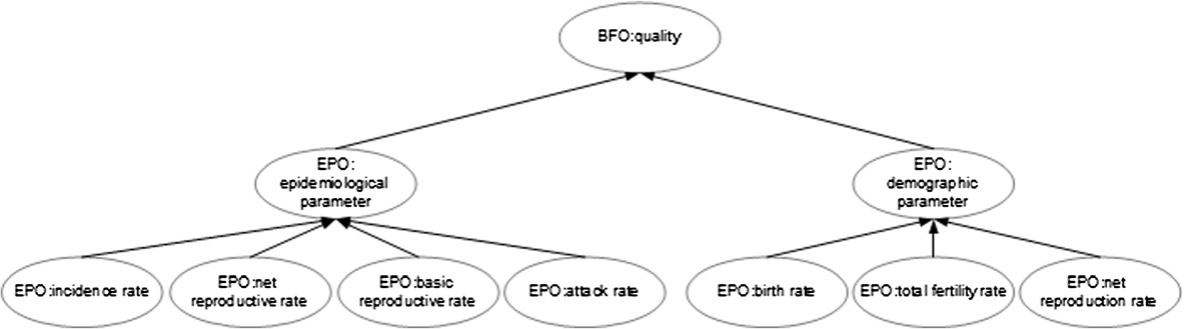
**A subgraph of EPO dedicated to epidemiological and demographic parameters.** This diagram represents a few classes of the epidemiological and demographic parameters branches of EPO, particularly some classes with similar labels.

**Figure 3:**
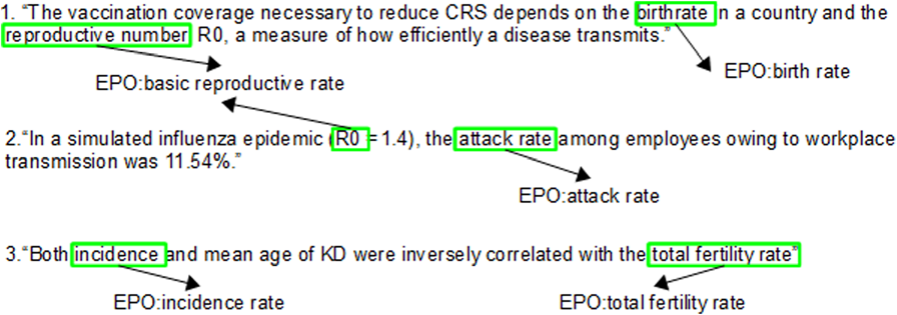
**Annotation of sentences from scientific papers with EPO classes.** This diagram exemplifies the usage of some of the EPO classes represented in Figure 4 to annotate entities mentioned in sentences extracted from scientific papers. ([1] Lessler J, Metcalf CJE, PLoS One 2013, 8, no. 7: e67639; [2] Kumar S et al., Am J Publ Heal 2013, 0: e1-e6.; [3] Nagao Y, PloS One 2013, 8, no. 7: e67934.)

The epidemiological and demographic parameters branches are, however, entirely composed of EPO classes. Figure 2 illustrates a portion of these branches, with their core classes and a few example subclasses, whose textual definitions are given in Table 3. Please note the potentially ambiguous classes ‘net reproduction rate’ and ‘net reproductive rate’ , the former a demographic parameter and the latter an epidemiological one, which illustrate the relevance of describing both parameter types in EPO. Figure 3 depicts the annotation of sentences extracted from scientific articles on epidemiology with EPO classes from the epidemiological and demographic parameters branches.

**Table 3:** **Textual definitions for classes in Figure**2

**Class label**	**Textual definition**
Epidemiological parameter	A parameter describing an epidemiological entity or event.
Demographic parameter	A parameter describing a demographic characteristic.
Incidence rate	The rate at which new events occur in a population. The numerator is the number of new events that occur in a defined period or other physical span. The denominator is the population at risk of experiencing the event during this period, sometimes expressed as person-time; it may instead be in other units, such as passenger-miles.
Net reproductive rate	In infectious disease epidemiology, the average number of secondary cases that will occur in a mixed host population of susceptibles and nonsusceptibles when one infected individual is introduced. Its relationship to the basic reproductive rate (R_0_) is given by R = R_0_x, where x is the proportion of the host population that is susceptible.
Basic reproductive rate	A measure of the number of infections produced, on average, by an infected individual in the early stages of an epidemic, when virtually all contacts are susceptible.
Attack rate	The proportion of a group that experiences the outcome under study over a given period (e.g., the period of an epidemic). This “rate” Â can be determined empirically by identifying clinical cases and/or by means of seroepidemiology. It also applies in noninfectious settings (e.g., mass poisonings). Because its time dimension is uncertain or arbitrarily decided, it should probably not be described as a rate.
Birth rate	A summary rate based on the number of live births in a population over a given period, usually 1 year.
Total fertility rate	The average number of children that would be born per woman if all women lived to the end of their childbearing years and bore children according to a given set of age-specific fertility rates. It is computed by summing the age-specific fertility rates for all ages and multiplying by the interval into which the ages are grouped. The TFR is an important fertility measure, providing the most accurate answer to the question “How many children does a woman have on average”.
Net reproduction rate	The average number of female children born per woman in a cohort subject to a given set of age-specific fertility rates, a given set of age specific mortality rates, and a given sex ratio at birth. This rate measures replacement fertility under given conditions of fertility and mortality: it is the ratio of daughters to mothers assuming continuation of the specified conditions of fertility and mortality. It is a measure of population growth from one generation to another under constant conditions. This rate is similar to the gross reproduction rate but takes into account that some women will die before completing their childbearing years. An NRR of 1.00 means that each generation of mothers is having exactly enough daughters to replace itself in the population.

### Applications

#### Epidemiological resource annotation

The EPO is integrated into NERO (Network of Epidemiology Related Ontologies), a collection of existing ontologies that supports the semantic annotation of epidemiology resources. NERO currently includes thirteen external ontologies and resources: MeSH (Medical Subject Headings vocabulary) [18], NCI Thesaurus [19], Disease Ontology [8], Infectious Disease Ontology [9], Symptom Ontology [10], Vaccine Ontology [11], Pathogen Transmission Ontology [10], Human Phenotype Ontology [23], Environment Ontology [24], ChEBI (Chemical Entities of Biological Interest) [25] and GeoPlanet™ [26].

NERO is integrated into the Epidemic Marketplace (EM) [27] (available at http://www.epimarketplace.net), a platform for sharing resources and knowledge within the Epidemiology community, which includes tools for the collection of epidemiological data through interoperable web services with other applications (e.g. from internet social networks [28], or from simulation results [2]). The EM allows users to browse a collection of semantically annotated epidemiology-related resources, including datasets, simulations and documents, and also to upload their own resources.

**Figure 4:**
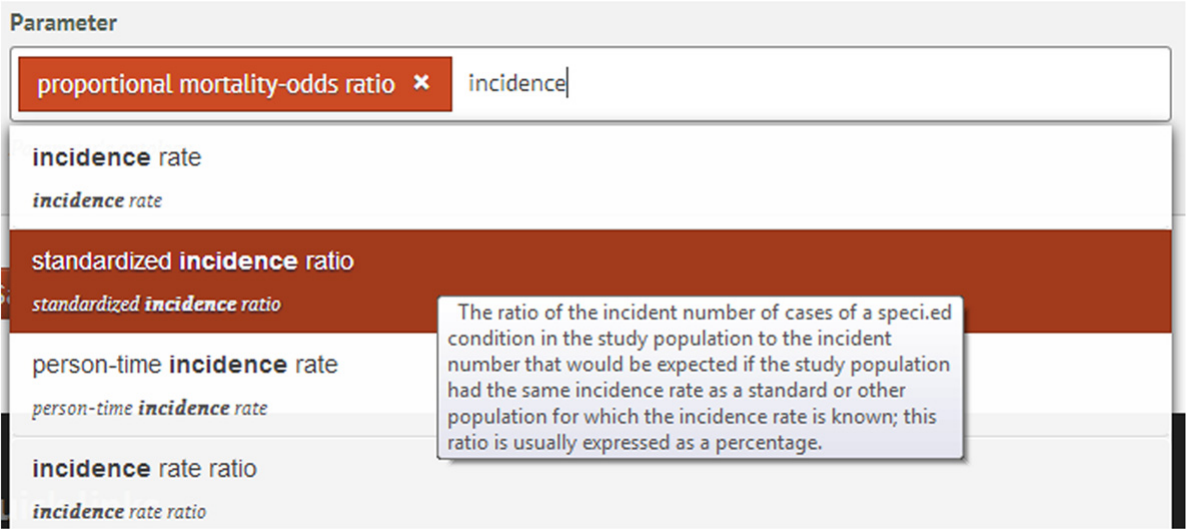
**Annotating an epidemiological resource with EPO using the online form of the Epidemic Marketplace.** The resource in this example is annotated with one EPO class, ‘proportional mortality odds-ratio’ , and another suitable class is being searched for by inputting the word ‘incidence’. The EM returns all entries in EPO with the word ‘incidence’ and the user can see their definitions in order to choose the best alternative.

Each EM resource is described with a set of metadata elements providing biological (e.g.: disease, symptom, host, vaccine, vector), geographical, environmental, demographical and epidemiological information as well as the associated time. To ensure a precise characterization, these metadata elements are filled-in with well-defined terms from NERO. Currently, the classes in EPO can be used in the metadata elements dedicated to transmission mode, demography and epidemiology. Figure 4 depicts the annotation of a resource on the EM online platform with an EPO class. Finding resources with specific epidemiological parameters can be of great use to epidemiology models and simulations that use these parameters as input to their systems.

Annotating epidemiology resources with EPO classes enables not only the specification of simple but precise queries that improve their retrieval rate, but also more complex knowledge discovery tasks, such as drawing inferences based on the semantics of these annotations [29].

#### Other applications

The EPO can also contribute beyond the scope of the Epidemic Marketplace. For instance, ontology-based text mining is a growing domain of interest for the biomedical literature, as evidenced by the increasing number of methods, resources and available initiatives [30]. The EPO can be used in conjunction with an ontology-based text mining approach to find relevant EPO terms in text [31,32].

EPO can also be a useful resource in ontology matching, particularly since it provides several cross-references to external resources. These have been shown to be particularly useful in the alignment of biomedical ontologies [33,34].

## Discussion and conclusions

EPO is an ontology that describes epidemiologically relevant concepts not well covered elsewhere. In conjunction with NERO, it aims at supporting the precise and comprehensive semantic annotation of epidemiology resources, such as documents, datasets, models and simulations. EPO aims at filling the gap of epidemiologically-specific terms that are missing from other ontologies, and consequently reuses many terms from OBO Foundry ontologies, such as IDO and TRANS. EPO is still in active development, and we expect it to grow considerably, particularly in the areas dedicated to epidemiology models, parameters and metrics. We are also considering an increase in granularity by reusing/linking to more specific ontologies, such as the Neglected Tropical Diseases Ontology [35]. We have initiated contacts with other OBO Foundry members, and hope to continue developing EPO in a collaborative effort. In particular, we expect EPO to be integrated into the mid-level Medical Surveillance Ontology, which is currently under development [36].

The annotation of epidemiology resources with EPO and other NERO ontologies answers the growing need to provide support for data integration and sharing in epidemiology. As more epidemiology resources are annotated both in the Epidemic Marketplace and elsewhere, the utility of EPO to the epidemiology community will continue to increase. The vast amounts of data currently locked in disparate datasets will become easily accessed and explored, and will help researchers to gain a better understanding of the transmission of infectious diseases in populations, and of the impact of public health measures and therapeutic approaches.

EPO, when combined with NERO in the Epidemic Marketplace platform, contributes to providing epidemiological researchers an effective framework for data integration and sharing.

## Methods

### Ontology building

EPO is being developed using Protégé 4.1 (http://protege.stanford.edu/), and encoded in OWL-DL (Web Ontology Language – Description Logic of the W3 Consortium). We chose OWL over OBO to take advantage of the many libraries and reasoners built for OWL, and specifically OWL-DL, to benefit from its support for class axioms, complete reasoning, inferences, and consistency-checking. Although we do not currently make use of all of these advantages, we expect EPO’s continued development to support complex queries in the context of its integration into the EM’s facilities. EPO is developed following the principles set by the OBO Foundry consortium. It uses the Basic Formal Ontology (BFO) as an upper-level ontology and the Information Artifact Ontology (IAO, http://purl.obolibrary.org/obo/iao) as a source for the annotation of properties. IAO has been adopted by many OBO foundry ontologies, such as IDO. Both BFO and IAO’s metadata portion are fully imported into EPO. In addition, EPO also uses relations imported from the OBO Relation Ontology [37]. All EPO classes contain textual definitions. Whenever possible, we added references to relevant external resources.

To ensure orthogonality, EPO imports classes from OBO candidate ontologies following the Minimal Information Reference External Ontology Term (MIREOT) strategy [38]. Although MIREOT is limited to source ontology URI, source term URI, and target direct superclass URI, we have also imported the label, to make the ontology more explicit to users and developers.

We plan to release new versions of EPO quarterly if required, for example to include the remaining dictionary entries that are not well-covered elsewhere. New releases of EPO will also be available for public use through the OBO Foundry repository and NCBO BioPortal.

### Knowledge acquisition

EPO was initially developed in a middle-out approach, where main entries found in the Dictionary of Epidemiology were specified into subclasses according to their extensive definitions, but were also generalized into BFO upper classes. The majority of relations between classes were derived from the definitions as well. Whenever possible, instead of creating novel classes based on dictionary entries (or in their specifications/generalizations) EPO imports the relevant classes from OBO ontologies and their subclasses. These belong to mostly two ontologies: the TRANS ontology for transmission of infection terms and IDO for transmission of infection participants and processes.

## Competing interests

The authors declare that they have no competing interests.

## Authors’ contributions

CP was responsible for the development of the ontology, including asserting the relations between all classes and editing textual definitions where needed, and wrote and edited the manuscript. JDF collaborated in the development of the ontology and was responsible for the integration of EPO in EM. FMC and MJS provided scientific direction and contributed to the development of the ontology. All authors critically reviewed and edited the manuscript. All authors read and approved the final manuscript.
